# Sensitivity and Stability Study of Test Conditions for a 1 kW Proton Exchange Membrane Fuel Cell Stack

**DOI:** 10.3390/membranes14090197

**Published:** 2024-09-18

**Authors:** Peng Xu, Yingmin Yi, Weijie Wang, Meng Xie, Yiwei Yuan

**Affiliations:** 1 School of Automation and Information Engineering, Xi’an University of Technology, Xi’an 710048, China; 2 XJ Electric Co., Ltd., Xuchang 461000, China; 3 Shanghai Qingneng Horizon New Energy Technology Co., Ltd., Shanghai 201900, China; xie.meng@horizonfuelcell.com

**Keywords:** proton exchange membrane fuel cell stack, assembly force, sensitivity of test conditions, optimal test parameters, stability

## Abstract

This study investigates the impact of compression force on stack performance and the effect of testing conditions on sensitivity of stack performance. It explores the variation of assembly force on the pressure distribution at different positions in a 1 kW proton exchange membrane fuel cell stack. Polarization curves and high-frequency resistance (HFR) changes of the stack were measured under different assembly forces, and the optimal assembly force of the stack was determined using the average single-cell voltage (HFR-free). The sensitivity of testing conditions was optimized, and the optimum test parameters at different current densities were identified within the selected range. Stack stability was tested at different current densities using the optimized test conditions, and the sensitivity of test conditions was verified by the fluctuation amplitude of single cell voltage and internal impedance.

## 1. Introduction

The proton exchange membrane fuel cell (PEMFC) converts chemical energy directly into electrical energy through an electrochemical reaction, with water as its only byproduct. As a result, PEMFCs are widely regarded as a clean and environmentally friendly energy source, which is of great significance in reducing greenhouse gas emissions and addressing climate change [1,2]. Compared to traditional combustion-based power generation methods, PEMFCs have higher energy conversion efficiency, enabling a larger proportion of the energy in a hydrogen fuel to be converted into usable electricity [3]. Furthermore, since PEMFCs primarily use hydrogen as fuel, they play a crucial role in promoting energy diversification, helping to reduce reliance on fossil fuels. Hydrogen can be produced through various pathways, including renewable energy sources, which further enhances energy security and sustainability [4]. A PEMFC consists of a series of individual cells, each composed of a membrane electrode assembly (MEA), bipolar plate (BPP), and sealing component [5]. The required number of single cells are stacked together and clamped by the front and rear end plates to form a complete fuel cell stack [6,7]. Within each cell, the catalyst in the MEA facilitates the oxygen reduction reaction (ORR) at the cathode and the hydrogen oxidation reaction (HOR) at the anode, converting the chemical energy of the reactants into electrical and thermal energy [8].

According to the process sequence, the end plates, collecting plates, insulation plates, BPP, and MEA are stacked neatly in correct order with the help of auxiliary positioning devices; appropriate clamping force is applied using a pressing platform, tightening them with screws, ties, or other methods to form a fuel cell stack [9]. Based on the principle of a fuel cell, the basic requirements for a stack assembly include the following [10,11]: ensuring that reactants are evenly distributed within the flow fields between and within the individual cells; maintaining appropriate compression of the gas diffusion layer (GDL) to ensure uniform contact between the GDL and BPP interfaces, which minimizes voltage loss; verifying that supporting structures, such as end plates, do not exhibit significant deformation and that there is no overall leakage within the stack; and ensuring that deformation caused by internal factors, such as temperature and humidity changes during operation, remains within an acceptable range [12]. The pressing force applied during assembly process of the stack has a significant impact on its performance [13,14]. On the one hand, it is necessary to apply as much pressure as possible to resist the stress changes caused by thermal deformation and expansion of internal components during the operation, ensuring airtightness between various components of the fuel cell stack and reducing the contact resistance at each interface [15]. On the other hand, excessive pressing force causes the GDL to be excessively compressed, resulting in a decrease in internal porosity and a greater impact of concentration polarization. The transfer of water and heat inside the stack will also be affected, increasing the risk of water flooding inside the electrodes [16]. Therefore, there is a reasonable value for the pressing force in assembling the stack, which can balance the overall sealing and obtain a smaller ohmic impedance. Simultaneously, the compression of the MEA should be controlled within an appropriate range to minimize concentration polarization and reduce the risk of water flooding in the electrodes. The varying characteristics of different components and materials inside fuel cell stacks necessitate different pressing forces for optimal performance across different stacks [17].

Changes in testing conditions for fuel cell stacks may cause problems with stack consistency and stability, meaning that a certain operating condition may have a relatively minor impact on the stack and may vary over a large range without affecting the overall performance and stability of the stack [18]. Conversely, the fuel cell stack may exhibit extreme sensitivity to changes in specific operating conditions, where even slight variations can decrease consistency and stability between cells, thereby affecting the overall performance output of the stack [19]. Common parameters influencing the operation of fuel cell stacks include the anode and cathode stoichiometric ratio, relative humidity, temperature, and gas pressure [20,21].

Therefore, we first examine the pressure distribution at various positions within the fuel cell stack. Then, we investigate how changes in assembly force affect the pressure distribution at these positions. The study involves testing the polarization curves and high-frequency resistance (HFR) of the stack under different assembly forces to determine the optimal assembly force based on the average single-cell voltage (HFR-free). The sensitivity of testing conditions for the stack is optimized, and the optimal testing parameters at each current density within the selected level range are identified. The stack stability is evaluated using these optimized testing conditions at different current densities, and the sensitivity of test conditions is verified by analyzing fluctuations in single-cell voltage and internal impedance. This experiment provides valuable insights and methodological advancements that can benefit future research and practical applications in this field.

## 2. Experimental

### 2.1. Pressure Distribution Testing at Different Positions of Fuel Cell Stack

**Figure 1:**
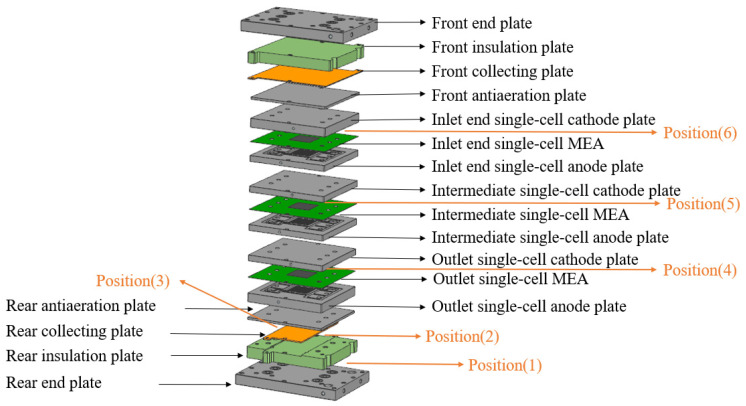
Schematic diagram of stack assembly structure and the placement of thin-film sensor of the pressure distribution test system.

The stack assembly utilized machine-carved graphite BPP, 316L stainless steel end plates, and 18 disc springs, and pressure plates were installed at the bottom. The stack was fastened with 8 screws along the long side and 4 screws along the short side. A thin-film pressure distribution system (SPI Tactilus H-series, USA) was employed to measure the assembly force at different positions within the stack. Three single cells and other components were assembled in sequence, with thin film sensors from the pressure distribution testing system placed at various positions within the stack (as shown in Figure 1). The pressure distribution at different interfaces inside the stack was recorded at 30, 35, 38, 40, 43, 45, 48, and 50 kN with a bolt tightening force of 1 N∙m. When loading pressure onto the stack, the pressure displacement hybrid control mode was adopted on the pressure mounting platform to fully release the internal stress; this ensured uniform pressure distribution inside the stack and prevented the BPP from sliding or cracking due to excessive pressure. The MEA had an active area of 340 cm^2^, and the catalyst layer was prepared using a slot coating and transfer printing process. A Freudenberg H24XC483 GDL and Gore M788.12 12 μm PEM were used for this experiment, with platinum loadings of 0.20 mg cm^−2^ (anode) and 0.40 mg cm^−2^ (cathode). The GDL and the coated catalytic membrane (CCM) were fixed by hot pressing treatment (TYC-3-S-PCD, TungYu (Ningbo) Hydraulic Machinery Co., Ltd., Ningbo, China). The MEA, stack, and components were manufactured and supplied by Henan Yuqing Power Co., Ltd., Xinxiang, China. The 3 single cells operated at a rated point of 1500 mA/cm^2^ and 0.65 V, with a rated output power of about 1 kW.

For convenience in descriptions, the following abbreviations are used: “anode” is abbreviated as An, “cathode” as Ca, “stoichiometric ratio” as Stoich. The subscript “a” represents the anode and subscript “c” represents the cathode.

During testing, the thin-film sensors of the pressure distribution testing system were sequentially placed at the following positions: between the rear end plate and the rear insulation plate (Position 1); between the rear insulation plate and the rear collector plate (Position 2); between the rear collector plate and the rear antiaeration plate (Position 3); between the outlet single-cell MEA and the outlet single-cell cathode plate (Position 4); between the intermediate single-cell MEA and the intermediate single-cell cathode plate (Position 5); and between the inlet single-cell MEA and the inlet single-cell cathode plate (Position 6) for repeated pressure distribution testing. Each position was tested repeatedly to analyze the force distribution at each contact interface under different pressing forces. After repositioning the thin-film sensors, the assembly conditions of the stack were maintained consistently with those of the initial setup.

### 2.2. Performance Testing of Fuel Cell Stack under Different Pressing Forces

The effect of different pressing forces on stack performance was compared and verified under varying pressure. The stack pressure force was gradually increased from 30 to 50 kN, with the bolt tightening force set at 1 N∙m. After the stack assembly was completed, a pressure holding test was conducted first.

Before performance testing, the stack was activated for 5 h under variable conditions, including 2 h of constant voltage and 30 min of constant current. The test conditions for the polarization curve were as follows: pressure (An/Ca) = 140:120 kPa, stoichiometric ratio (An/Ca) = 1.5/1.8, relative humidity (An/Ca) = 60:60 RH %, and the stack temperature was controlled at 76 °C.

During the testing, the high-frequency resistance (HFR) was used to represent the stack’s ohmic impedance, which includes proton and electron transmission impedance as well as the interface impedance of stack components. The proton transport impedance of the MEA accounted for the main part, and the transmission impedance was mainly affected by the stack’s relative humidity. Electrochemical impedance spectroscopy (EIS) was conducted under the same test conditions as the polarization curve, at constant current under the corresponding current density. The perturbation current was set at 2.5% of the working current, and the scanning frequency ranged from 0.1 to 1000 Hz.

### 2.3. Stack Performance Sensitivity Optimization Testing

Sensitivity optimization testing for stack test conditions was conducted using an orthogonal method. For 0.30 A cm^−2^, 3 factors and 2 levels were selected, and an L4 orthogonal table was used. For current densities of 0.70, 1.20, and 1.60 A cm^−2^, 4 factors and 3 levels were selected, using an L9 orthogonal table. A pressure parallel testing was added for each current density to verify the pressure sensitivity at that point. The cooling water flow rate was maintained between 3 and 6 L min^−1^, and the stack temperature was controlled between 65 and 72 °C. A total of 35 experiments were conducted. The specific experimental design is detailed in Table 1 and Table 2.

An intuitive analysis method was used to evaluate the test results. This approach involved summing up the test results of each factor at the same level and calculating the average value. The test results corresponding to each level of each factor were then determined based on these average values. The difference in the average value across factor levels indicated the sensitivity of the test results to each factor. After stabilizing each test parameter under the specified conditions, the system was operated at a constant current for 30 min and voltage data (average value within 5 s) were recorded. Following shutdown and purge, the next set of tests was conducted. At each given current density, the stability of the output voltage and internal resistance during the operation was used to represent the stability of the internal reaction state and power generation capacity of the stack. Using the optimized testing conditions, stability tests on output voltage and internal impedance of the stack were conducted at different current densities to verify the sensitivity of test conditions to the output results.

## 3. Results and Discussion

### 3.1. Analysis of Pressure Distribution Test Results at Different Positions

The film sensor of the pressure distribution test system was sequentially placed at 6 different positions within the stack. For each applied pressure force—30, 35, 38, 40, 43, 45, 48, and 50 kN—the pressure distribution of various positions in the stack was recorded. The summarized pressure distribution results for these different pressures are shown in Table 3.

The test results show that, under the same pressure, the stress conditions at each interface at different positions of the stack are largely consistent, with no significant deviation, as indicated by vertical data comparisons. This suggests that the flatness of each component meets the requirements of the stack, and there is no internal pressure concentration. As the pressure force increases, the pressure value at the same position shows an upward trend, although the increase is modest, as indicated by horizontal data comparisons. This indicates that pressure force within this range is approaching the deformation limit of the components. Further increasing the pressure force has minimal effect on pressure changes between components, and may risk overpressure damage to the components, such as graphite bipolar plate cracking or slipping, etc.

### 3.2. Analysis of Stack Performance Test Results for Different Pressures

Before conducting the polarization curve test, a stack pressure holding test was carried out. The initial pressure of 210 kPa was held for 5 min and then reduced to 203 kPa. Similarly, the initial pressure of 50 kPa was maintained in the hydrogen cavity for 5 min before dropping to 48 kPa. The hydrogen crossover flow was 0.6 mL min^−1^. and the water cavity leakage was 0.2 mL min^−1^. The results of the pressure holding test met the stack assembly requirements.

**Figure 2:**
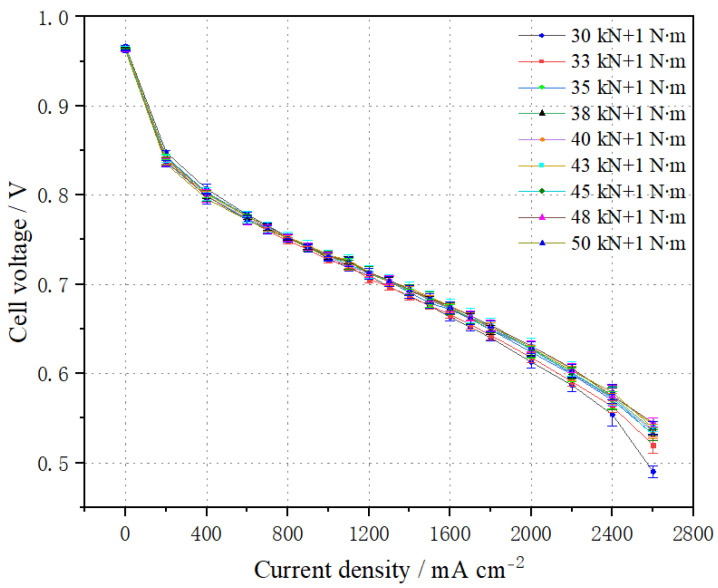
Comparison of polarization curves of the stack at different pressures.

Stack performance was tested at different pressures, with the results shown in Figure 2. The pressure force and bolt tightening force are expressed as “kN + N∙m” in the following: for example, a stack assembled with a pressure force of 30 kN and a bolt tightening force of 1 N∙m is marked as 30 kN + 1 N∙m.

From Figure 2, it can be observed that at the same current density, the average voltage of the cell initially increases with increasing pressure, but eventually decreases due to excessive pressure. For instance, at a current density of 1.0 A cm^−2^, the average cell voltage reaches 0.733 V with a pressure force of 43 kN + 1 N∙m. However, when the pressure exceeds 43 kN + 1 N∙m, the stack performance begins to decrease. At a pressure of 50 kN + 1 N∙m, the average cell voltage at 1.0 A cm^−2^ is 0.731 V, representing a decrease of 2 mV. In the low current density range, the contact resistance significantly affects stack performance, and it decreases notably with increased pressure. Conversely, at high current densities, increased pressure weakens gas transmission, causing a slight decrease in stack performance. This is because excessive pressure leads to MEA overpressure, reducing the permeability of the GDL, and thereby decreasing the effective diffusion rate and permeability of the reaction gas. This results in higher gas transfer impedance and a reduction in stack performance at high current densities.

**Figure 3:**
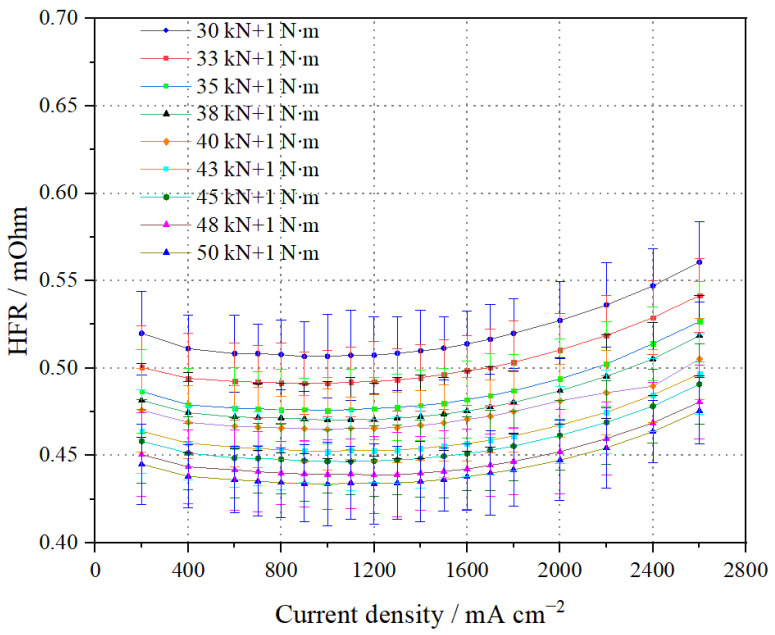
Comparison of high-frequency resistance (HFR) test results of the stack at different pressures.

The HFR test results of the stack at different pressures are shown in Figure 3. The stack’s HFR gradually decreases with increasing pressing force due to the significant reduction in interface contact resistance. When the pressure force reaches 50 kN + 1 N∙m, the HFR at 1.0 A cm^−2^ is the lowest, at 0.434 mΩ, and similarly, the HFR at 2.0 A/cm^2^ is also the lowest, at 0.447 mΩ. However, it can also be observed that, with an increase in current density (great than 0.80 A cm^−2^), the HFR exhibits a slight increasing trend. This is because the gas flow required for the stack increases with the current density. Although the amount of water generated also increases, more water is carried away by the reaction gas, leading to a decrease in the PEM water content. This reduction in water content impairs the membrane’s proton conductivity, resulting in a corresponding increase in HFR.

**Figure 4:**
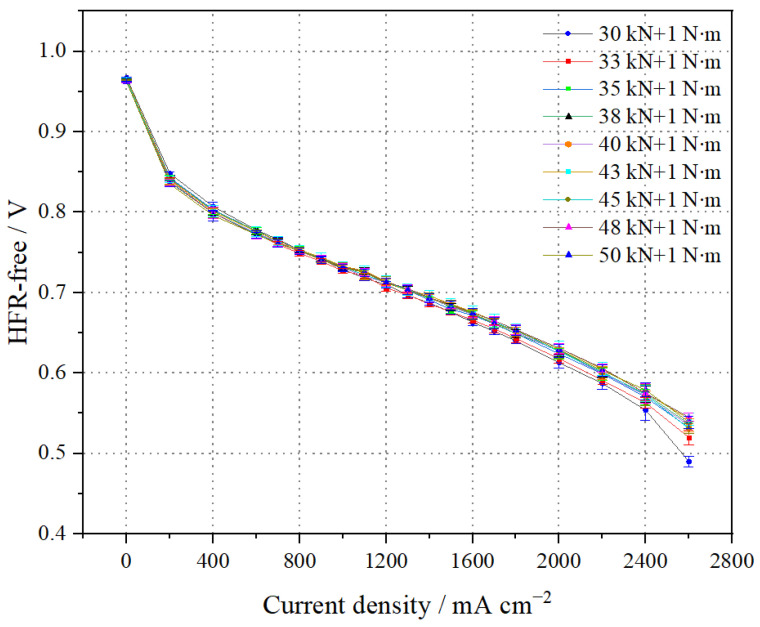
Comparison of polarization curves of the stack at different pressures (HFR-free).

The stack polarization curves (HFR-free) at different pressure forces are shown in Figure 4. It can be seen that at 1.0 A cm^−2^, the average single-cell voltage (HFR-free) is 0.785 V when the pressure force is 43 kN + 1 N∙m, while at 2.0 A cm^−2^, it is 0.736 V. At 1.0 A cm^−2^, the average single-cell voltage (HFR-free) is 0.780 V when the pressure force is 50 kN + 1 N∙m, and it is 0.730 V at 2.0 A cm^−2^. The results indicate that, below a pressure force of 43 kN + 1 N·m, the average single-cell voltage (HFR-free) decreases with increasing pressure force. However, once the pressure force exceeds 43 kN + 1 N·m, the voltage begins to decline. This is due to the increased compression of the GDL, which can become embedded in the grooves of the bipolar plates and affect gas transport flux. Excessive compression of the GDL also reduces its gas transport capacity. Therefore, the optimal assembly force for the stack in this experiment is determined to be 43 kN + 1 N·m.

### 3.3. Analysis of Stack Test Results of Test Condition Sensitivity

According to the test design, sensitivity tests for stack test conditions were conducted individually. The results are summarized in Table 4.

(1) Sensitivity analysis of the stack performance at 0.30 A cm^−2^

**Figure 5:**
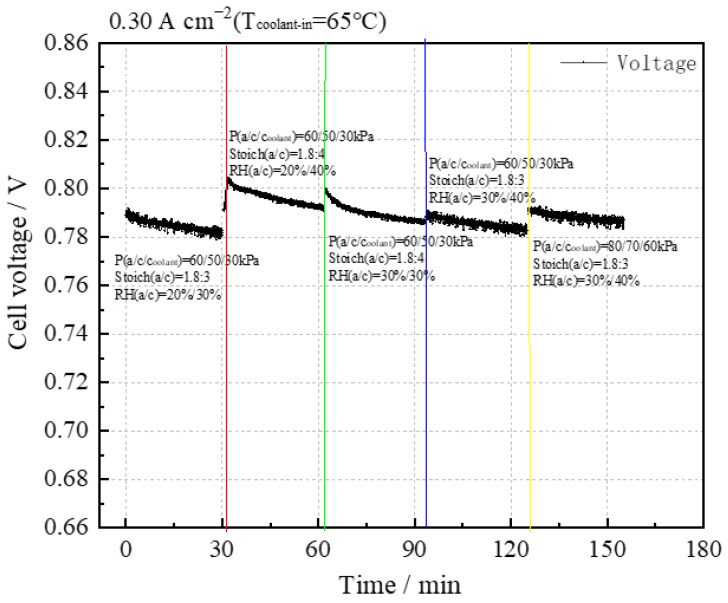
Sensitivity analysis of stack performance at 0.30 A cm^−2^.

The sensitivity test results at 0.30 A cm^−2^ are illustrated in Figure 5. It can be seen that under the conditions of P(a/c/coolant) = 60/50/30 kPa, the stack performance improves with increases in RH_c_ and Stoich(c), and the stack performance is highest under the conditions of RH_c_ = 40% and Stoich(c) = 4. When P(a/c/coolant) = 60/50/30 kPa, and the RH_a_ increases to 30%, there is no significant improvement in stack performance.

The intuitive analysis method was employed to evaluate the test results, leading to the creation of tables summarizing stack performance and consistency at a current density of 0.30 A cm^−2^, as detailed in Table 5 and Table 6.

According to the mean value, when RH_a_:RH_c_ = 20%:40% and Stoich.(c) ratio = 4, the stack performance is the highest.

It was concluded from the range that the cathode stoichiometric ratio has a great influence on the stack performance within the selected level range.

From the mean values, it can be concluded that the performance consistency at this current density is minimally affected by the experimental conditions.

The results indicate that all three factors have a similar impact on stack performance consistency within the selected range of levels.

Comparing the results from experiments 4 and 5, it is observed that increasing the reaction gas pressure for both anode and cathode slightly improves stack performance.

In summary, higher cathode humidity and stoichiometric ratio positively affect stack performance, with better performance and consistency achieved at higher values. Additionally, increasing pressure enhances stack performance.

Therefore, at a current density of 0.30 A cm^−2^ within the selected level, the optimal test parameters are the following: P(a/c) = 60:50 kPa, T (inlet water) = 65 °C, Stoich(a/c) = 1.5:4, and RH(a/c) = 20%:40%.

(2) Sensitivity analysis of the stack performance at 0.70 A cm^−2^

**Figure 6:**
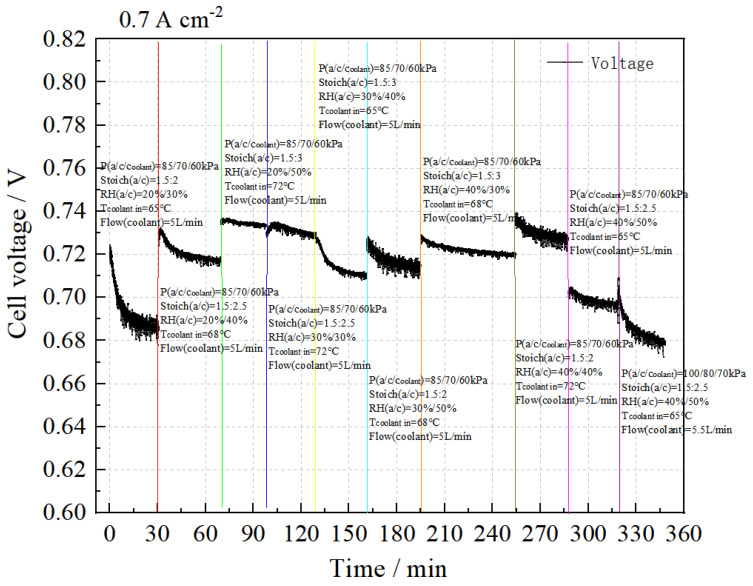
Sensitivity analysis of stack performance at 0.70 A cm^−2^.

The performance sensitivity test results at 0.70 A cm^−2^ are presented in Figure 6. It can be observed that, under the conditions of P(a/c/coolant) = 85/70/60 kPa, the stack performance improves with increases in the cooling water temperature and cathode stoichiometric ratio, and optimal performance is achieved at RH_a_:RH_c_ = 40%:40%, cooling water temperature 72 °C, and Stoich(a/c) = 1.5:3. Conversely, when P(a/c/coolant) = 100/80/70 kPa, with a cooling water temperature of 65 °C and Stoich(a/c) = 1.5:2.5, the stack performance shows a significant decrease.

The intuitive analysis method was employed to evaluate the test results, resulting in the analysis tables for stack performance and consistency at a current density of 0.70 A cm^−2^, as shown in Table 7 and Table 8.

Based on the mean values, the stack performance is highest when RH_a_:RH_c_ = 40%:40%, Stoich.(c) ratio = 3, and cooling water temperature 72 °C.

The results indicate that the cooling water temperature has the most significant impact on stack performance, while the relative humidity has a minimal effect.

According to the mean values, the stack consistency is significantly affected by the cooling water temperature within the selected level range.

The results show that the cooling water temperature has a major influence on stack consistency, while anode relative humidity and cathode stoichiometric ratio have minimal impact within the selected levels.

Compared to the experimental results of 14/15 #, increasing gas pressure does not significantly improve stack performance.

In summary, within the selected level range, higher anode relative humidity and cooling water temperature lead to better stack performance. At the current density of 0.70 A cm^−2^, the optimal test parameters are P(a/c) = 85:70 kPa, T (water inlet) = 72 °C, Stoich(a/c) = 1.5:3, and RH(a/c) = 40%: 40%.

(3) Sensitivity analysis of the stack performance at 1.20 A cm^−2^

**Figure 7:**
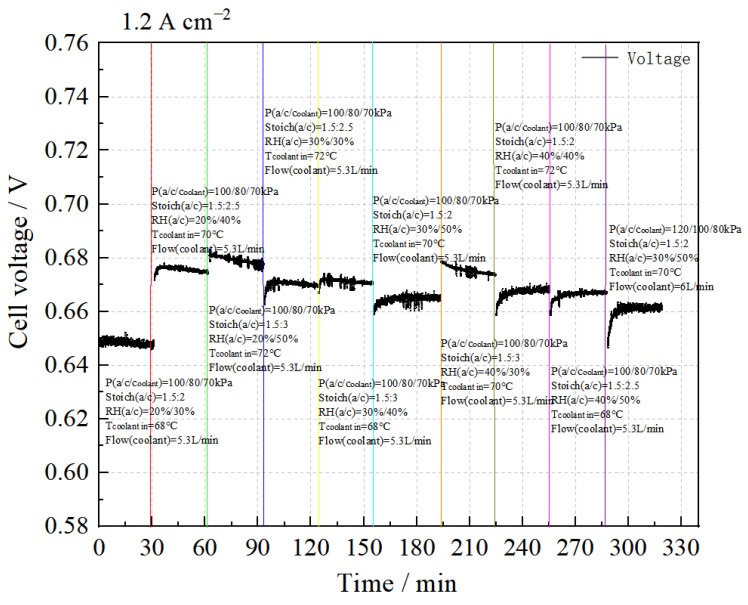
Sensitivity analysis of stack performance at 1.20 A cm^−2^.

The performance sensitivity test results at a condition of 1.20 A cm^−2^ are shown in Figure 7. The data indicate that under the condition P(a/c/coolant) = 100/80/70 kPa, stack performance improves with increased cooling water temperature, cathode stoichiometric ratio, and relative humidity. The highest stack performance is achieved with a cooling water temperature of 72 °C, Stoich(a/c) = 1.5:3, and RH(a/c) = 20%/50%. Conversely, when the water temperature is 70 °C, Stoich(a/c) = 1.5:2, RH(a/c) = 30%/50%, and P(a/c/coolant) = 120/100/80 kPa, the stack performance decreases.

The intuitive analysis method was used to analyze the test results and the analysis tables for stack performance and consistency at a current density of 1.20 A cm^2^ are shown in Table 9 and Table 10.

According to the mean values, the stack performance is highest when RH_a_:RH_c_ = 40%:50%, Stoich.(c) ratio = 3, and cooling water temperature = 72 °C.

We conclude that, within the selected level range, the cathode stoichiometric ratio and cooling water temperature have a significant impact on stack performance at 1.20 A cm^−2^.

According to the mean value, the stack performance is highest when RH_a_ = 20% or 30%, RHc = 50%, cathode stoichiometric ratio = 2.5, and cooling water temperature = 70 °C or 72 °C.

The results show that at 1.20 A cm^−2^, the cathode stoichiometric ratio and cooling water temperature notably influence stack consistency.

However, compared with the experimental results of 21/25 #, increasing gas pressure does not significantly improve stack performance.

The optimal test parameters within the selected level range are P(a/c) = 100:80 kPa, T (water inlet) = 72 °C, Stoich(a/c) = 1.5:3, and RH(a/c) = 40%: 40%.

(4) Sensitivity analysis of the stack performance at 1.60 A cm^−2^

**Figure 8:**
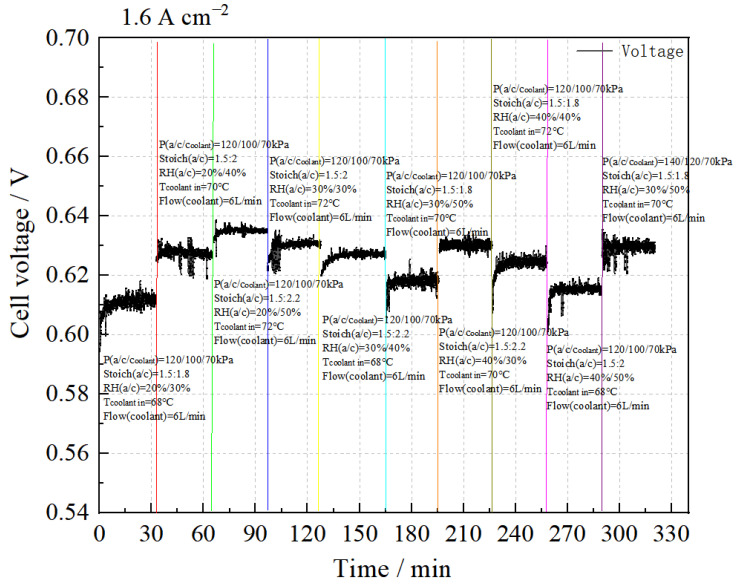
Sensitivity analysis of stack performance at 1.60 A cm^−2^.

The test results at 1.60 A cm^−2^ are shown in Figure 8. It can be seen that under the condition P(a/c/coolant) = 120/100/70 kPa, the stack performance increases with increasing cooling water temperature, cathode stoichiometric ratio, and relative humidity. The optimal performance is achieved under the conditions of cooling water temperature = 72 °C, Stoich(a/c) = 1.5:2.2, and RH(a/c) = 20%/50%. When the cooling water temperature is reduced to 70 °C, Stoich(a/c) = 1.5:2, and RH(a/c) = 30%/50%, increasing the back pressure to P(a/c/coolant) = 140/120/70 kPa leads to a significant improvement in stack performance.

The intuitive analysis method was applied to evaluate the test results, resulting in the analysis tables of stack performance and consistency at a current density of 1.60 A cm^−2^, as shown in Table 11 and Table 12.

According to the mean values, the stack achieves its highest performance when RH_a_:RH_c_ = 30%:40%, Stoich.(c) ratio = 2.2, and cooling water temperature = 72 °C.

The analysis indicates that the cathode stoichiometric ratio significantly impacts stack performance within the selected level range.

Based on the mean value analysis, the stack demonstrates optimal consistency when RH_a_:RH_c_ = 30%:30%, cathode stoichiometric ratio = 2.2, and cooling water temperature = 72 °C.

The results show that within the selected level range at 1.60 A cm^−2^, both the stoichiometric ratios of the cathode and anode significantly impact stack consistency, while cathode relative humidity has a minimal effect.

In comparison with the experimental results of tests 31/35 #, it is evident that increasing gas pressure notably enhances both the performance and consistency of the stack.

In summary, within the selected level range, as anode humidity increases, the stack performance initially rises and then declines, with consistency proportional to performance. The stack achieves optimal performance and consistency at RH_a_ = 30%. The effect of cathode humidity on stack performance is the same as that of the anode, but it does not impact consistency. Higher cathode stoichiometric ratios lead to improved stack performance and consistency. Similarly, an increase in cooling water temperature enhances both stack performance and consistency. Additionally, higher gas pressure results in better performance and consistency.

Therefore, at 1.60 A cm^−2^ within the selected level range, the optimal test parameters are P(a/c) = 120:100 kPa, T (water inlet) = 72 °C, Stoich(a/c) = 1.5:2.2, and RH(a/c) = 30%: 40%.

(5) Summary of optimal test conditions for the stack

In the selected level range, the optimal test parameters for the stack at various current densities are summarized in Table 13.

Based on the above conditions, improved stack performance and consistency can be achieved. However, it is necessary to further verify whether the stack performance can be maintained under these test conditions.

### 3.4. Stability Verification of Stack under the Optimal Test Conditions

**Figure 9:**
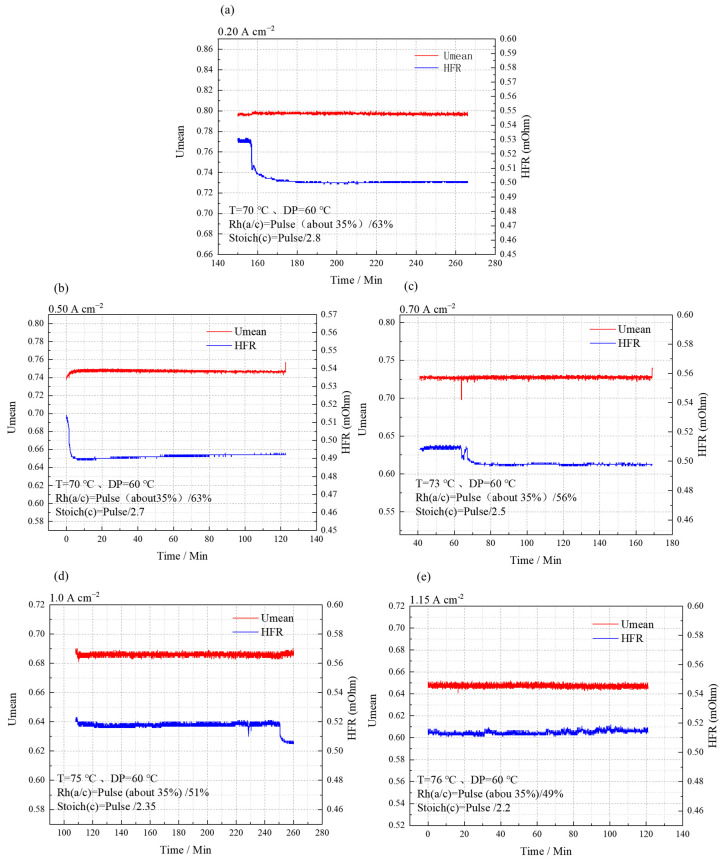
Voltage stability results at 0.20 (**a**)/0.50 (**b**)/0.70 (**c**)/1.00 (**d**)/1.15 (**e**) A cm^−2^.

The optimal test parameters were used to assess stack stability at current densities of 0.20, 0.50, 0.70, 1.00, and 1.15 A cm^−2^. The results, as shown in Figure 9, indicate that at each current density, the voltage fluctuation between single cells is within ±2 mV (decreasing), and the stack’s internal resistance, represented by the HFR, remains stable at 0.5 ± 0.02 mΩ. This demonstrates that the condition sensitivity test results ensure stack stability across the different current densities.

## 4. Conclusions

(1) Under the same pressure conditions, the interface forces remain consistent with no significant deviations. As the pressure force increases, the pressure value at each position trends upwards, though the increase is relatively modest. At the same current density, the average voltage of the cell initially increases with increasing pressure, but eventually decreases due to excessive pressure. The optimal assembly force for the experimental stack is determined to be 43 kN + 1 N∙m.
(2) At low current densities, contact resistance has a greater impact on stack performance compared to gas transmission, and contact resistance decreases significantly with increasing pressure force. At high current densities, increasing pressure force weakens gas transfer ability, leading to a slight decrease in stack performance. The internal resistance of the stack, as indicated by the HFR, decreases with increasing pressure force, while the HFR itself increases with higher current density.
(3) The orthogonal method was employed to optimize the sensitivity of test conditions, leading to the determination of the optimal test parameters for each current density of the experimental stack. Stability testing of the stack’s output voltage and internal impedance was conducted using these optimized conditions to assess the sensitivity of the output results. It was observed that the voltage fluctuation between individual cells was within ±2 mV (decreasing), and the internal resistance, as indicated by the HFR, remained stable at 0.5 ± 0.02 mΩ. These findings demonstrate that the sensitivity analysis ensures stable stack performance across different current densities.

## Figures and Tables

**Table 1:** Sensitivity testing orthogonal experimental design.

Current Density (A cm^−2^)	Pressure (A/C, kPa)	RH_a_ (%)	RH_c_ (%)	Anode Stoich.	Cathode Stoich.	Orthogonal Table	Supplementary Pressure Testing (A/C, kPa)
0.30	60/50	20	30	1.8	3	L4(23)	80/70
		30	40		4		
0.70	85/70	20	30	1.5	2	L9(34)	100/80
		30	40		2.5		
		40	50		3		
1.20	100/80	20	30	1.5	2	L9(34)	120/100
		30	40		2.5		
		40	50		3		
1.60	120/100	20	30	1.5	1.8	L9(34)	140/120
		30	40		2		
		40	50		2.2		

**Table 2:** Sensitivity test parameters.

**0.30 A cm^−2^:**							
**No.**	**Pressure** **(a/c/Coolant, kPa)**	**Stoich.(a/c)**	**Inlet Water Temperature (°C)**	**RH_a_ (%)**	**RH_c_ (%)**	**Flow Rate of Cooling Water (L min^−1^)**	**Run Time (min)**
1	60/50/30	1.8/3	65	20	30	3	30
2	60/50/30	1.8/4	65	20	40		30
3	60/50/30	1.8/4	65	30	30		30
4	60/50/30	1.8/3	65	30	40		30
5	80/70/60	1.8/3	65	30	40	5	30
**0.70 A cm^−2^:**							
**No.**	**Pressure** **(a/c/Coolant, kPa)**	**Stoich.(a/c)**	**Inlet Water Temperature (°C)**	**RH_a_ (%)**	**RH_c_ (%)**	**Flow Rate of Cooling Water (L min^−1^)**	**Run Time (min)**
6	85/70/60	1.5/2	65	20	30	5	30
7	85/70/60	1.5/2.5	68	20	40		30
8	85/70/60	1.5/3	72	20	50		30
9	85/70/60	1.5/2.5	72	30	30		30
10	85/70/60	1.5/3	65	30	40		30
11	85/70/60	1.5/2	68	30	50		30
12	85/70/60	1.5/3	68	40	30		30
13	85/70/60	1.5/2	72	40	40		30
14	85/70/60	1.5/2.5	65	40	50		30
15	100/80/70	1.5/2.5	65	40	50	5.3	30
**1.20 A cm^−2^:**							
**No.**	**Pressure** **(a/c/Coolant, kPa)**	**Stoich.(a/c)**	**Inlet Water Temperature (°C)**	**RH_a_ (%)**	**RH_c_ (%)**	**Flow Rate of Cooling Water (L min^−1^)**	**Run Time (min)**
16	100/80/70	1.5/2	68	20	30	5.3	30
17	100/80/70	1.5/2.5	70	20	40		30
18	100/80/70	1.5/3	72	20	50		30
19	100/80/70	1.5/2.5	72	30	30		30
20	100/80/70	1.5/3	68	30	40		30
21	100/80/70	1.5/2	70	30	50		30
22	100/80/70	1.5/3	70	40	30		30
23	100/80/70	1.5/2	72	40	40		30
24	100/80/70	1.5/2.5	68	40	50		30
25	120/100/80	1.5/2	70	30	50	6	30
**1.60 A cm^−2^:**							
**No.**	**Pressure (a/c/Coolant, kPa)**	**Stoich.(a/c)**	**Inlet Water Temperature (°C)**	**RH_a_ (%)**	**RH_c_ (%)**	**Flow Rate of Cooling Water (L min^−1^)**	**Run Time (min)**
26	120/100/70	1.5/1.8	68	20	30	6	30
27	120/100/70	1.5/2	70	20	40		30
28	120/100/70	1.5/2.2	72	20	50		30
29	120/100/70	1.5/2	72	30	30		30
30	120/100/70	1.5/2.2	68	30	40		30
31	120/100/70	1.5/1.8	70	30	50		30
32	120/100/70	1.5/2.2	70	40	30		30
33	120/100/70	1.5/1.8	72	40	40		30
34	120/100/70	1.5/2	68	40	50		30
35	140/120/70	1.5/2	70	30	50		30

**Table 3:** Pressure distributions at different positions and pressure forces of the stack (unit: Bar).

	Pressure Force	30 kN	33 kN	35 kN	38 kN	40 kN	43 kN	45 kN	48 kN	50 kN
Position of Pressure Distribution Test System										
Position 1		13.24	13.33	13.42	13.49	13.56	13.61	13.71	13.78	13.72
Position 2		13.29	13.34	13.43	13.53	13.62	13.67	13.69	13.73	13.77
Position 3		13.47	13.53	13.56	13.61	13.64	13.67	13.77	13.72	13.76
Position 4		13.45	13.52	13.57	13.62	13.66	13.69	13.79	13.74	13.77
Position 5		13.24	13.35	13.42	13.49	13.56	13.61	13.74	13.78	13.78
Position 6		13.29	13.37	13.43	13.53	13.62	13.67	13.74	13.78	13.78

**Table 4:** The stack test condition sensitivity test results.

**0.30 A cm^−2^**													
**No.**	**Pressure (a/c/Coolant, kPa)**	**Stoich.(a/c)**	**Cooling Water Temperature (°C)**	**RH_a_** **(%)**	**RH_c_** **(%)**	**Water Flow Rate (L min^−1^)**	**Run Time** **(min)**	**Remark**	**Cold End Cell (V)**	**Middle Cell (V)**	**Hot End Cell (V)**	**Mean Value (V)**	**HFR (mΩ)**
1	60/50/30	1.8/3	65	20	30	3	30		0.790	0.778	0.777	0.782	0.691
2	60/50/30	1.8/4	65	20	40	3	30		0.797	0.791	0.789	0.792	0.593
3	60/50/30	1.8/4	65	30	30	3	30		0.789	0.787	0.784	0.787	0.730
4	60/50/30	1.8/3	65	30	40	3	30		0.787	0.783	0.780	0.783	0.568
5	80/70/60	1.8/3	65	30	40	5	30	Pressure supplement experiment for 4#	0.789	0.786	0.783	0.786	0.560
**0.70 A cm^−2^**													
**No.**	**Pressure (a/c/coolant, kPa)**	**Stoich.(a/c)**	**Cooling water temperature (°C)**	**RH_a_** **(%)**	**RH_c_** **(%)**	**Water flow rate (L min^−1^)**	**Run time** **(min)**	**Remark**	**Cold end cell (V)**	**Middle cell (V)**	**Hot end cell (V)**	**Mean value (V)**	**HFR (mΩ)**
6	85/70/60	1.5/2	65	20	30	5	30		0.701	0.691	0.668	0.687	0.516
7	85/70/60	1.5/2.5	68	20	40	5	30		0.703	0.721	0.708	0.711	0.527
8	85/70/60	1.5/3	72	20	50	5	30		0.735	0.734	0.732	0.734	0.548
9	85/70/60	1.5/2.5	72	30	30	5	30		0.734	0.727	0.726	0.729	0.570
10	85/70/60	1.5/3	65	30	40	5	30		0.725	0.713	0.693	0.710	0.519
11	85/70/60	1.5/2	68	30	50	5	30		0.718	0.716	0.705	0.713	0.501
12	85/70/60	1.5/3	68	40	30	5	30		0.729	0.726	0.713	0.723	0.546
13	85/70/60	1.5/2	72	40	40	5	30		0.727	0.728	0.726	0.727	0.517
14	85/70/60	1.5/2.5	65	40	50	5	30		0.708	0.700	0.683	0.697	0.491
15	100/80/70	1.5/2.5	65	40	50	5.3	30	Pressure supplement experiment for 14#	0.693	0.683	0.661	0.679	0.484
**1.20 A cm^−2^**													
**No.**	**Pressure** **(a/c/coolant, kPa)**	**Stoich.(a/c)**	**Cooling water temperature (°C)**	**RH_a_** **(%)**	**RH_c_** **(%)**	**Water flow rate (L min^−1^)**	**Run time** **(min)**	**Remark**	**Cold end cell (V)**	**Middle cell (V)**	**Hot end cell (V)**	**Mean value (V)**	**HFR (mΩ)**
16	100/80/70	1.5/2	68	20	30	5.3	30		0.649	0.659	0.634	0.647	0.511
17	100/80/70	1.5/2.5	70	20	40	5.3	30		0.674	0.676	0.673	0.647	0.522
18	100/80/70	1.5/3	72	20	50	5.3	30		0.679	0.676	0.679	0.678	0.542
19	100/80/70	1.5/2.5	72	30	30	5.3	30		0.670	0.670	0.669	0.670	0.554
20	100/80/70	1.5/3	68	30	40	5.3	30		0.673	0.674	0.665	0.671	0.523
21	100/80/70	1.5/2	70	30	50	5.3	30		0.663	0.670	0.664	0.666	0.499
22	100/80/70	1.5/3	70	40	30	5.3	30		0.674	0.673	0.674	0.674	0.547
23	100/80/70	1.5/2	72	40	40	5.3	30		0.667	0.672	0.665	0.668	0.510
24	100/80/70	1.5/2.5	68	40	50	5.3	30		0.667	0.671	0.663	0.667	0.494
25	120/100/80	1.5/2	70	30	50	6	30	Pressure supplement experiment for 21#	0.663	0.669	0.654	0.662	0.485
**1.60 A cm^−2^**													
**No.**	**Pressure** **(a/c/coolant, kPa)**	**Stoich.(a/c)**	**Cooling water temperature (°C)**	**RH_a_** **(%)**	**RH_c_** **(%)**	**Water flow rate (L min^−1^)**	**Run time** **(min)**	**Remark**	**Cold end cell (V)**	**Middle cell (V)**	**Hot end cell (V)**	**Mean value (V)**	**HFR (mΩ)**
26	120/100/70	1.5/1.8	68	20	30	6	30		0.616	0.622	0.599	0.612	0.496
27	120/100/70	1.5/2	70	20	40	6	30		0.629	0.635	0.615	0.626	0.510
28	120/100/70	1.5/2.2	72	20	50	6	30		0.636	0.640	0.629	0.635	0.511
29	120/100/70	1.5/2	72	30	30	6	30		0.629	0.635	0.628	0.631	0.513
30	120/100/70	1.5/2.2	68	30	40	6	30		0.628	0.635	0.626	0.630	0.494
31	120/100/70	1.5/1.8	70	30	50	6	30		0.615	0.629	0.611	0.618	0.491
32	120/100/70	1.5/2.2	70	40	30	6	30		0.630	0.638	0.623	0.630	0.493
33	120/100/70	1.5/1.8	72	40	40	6	30		0.624	0.634	0.618	0.625	0.495
34	120/100/70	1.5/2	68	40	50	6	30		0.616	0.625	0.605	0.615	0.481
35	140/120/70	1.5/1.8	70	30	50	6	30	Pressure supplement experiment for 31#	0.625	0.639	0.626	0.630	0.482

**Table 5:** The intuitive analysis table of stack performance at 0.30 A cm^−2^.

Column	1	2	3	
Factor	RH_a_	RH_c_	Stoich.(c)	Stack Performance (V)
Experiment 1	1(20%)	1(30%)	1(3)	0.782
Experiment 2	1	2(40%)	2(4)	0.792
Experiment 3	2(30%)	1	2	0.787
Experiment 4	2	2	1	0.783
Mean value 1	0.787	0.785	0.783	
Mean value 2	0.785	0.788	0.790	
Sample Range	0.002	0.003	0.007	

**Table 6:** The intuitive analysis table of stack consistency at 0.30 A cm^−2^.

Column	1	2	3	
Factor	RH_a_	RH_c_	Stoich.(c)	Standard Deviation (SD)
Experiment 1	1(20%)	1(30%)	1(3)	0.007
Experiment 2	1	2(40%)	2(4)	0.004
Experiment 3	2(30%)	1	2	0.003
Experiment 4	2	2	1	0.004
Mean value 1	0.006	0.005	0.006	
Mean value 2	0.004	0.004	0.004	
Sample Range	0.002	0.001	0.002	

**Table 7:** The intuitive analysis table of stack performance at 0.70 A cm^−2^.

Column	1	2	3	4	
Factor	RH_a_	RH_c_	Stoich. (c)	T (Inlet Water) (°C)	Stack Performance (V)
Experiment 6	1(20%)	1(30%)	1(2.0)	1(65)	0.687
Experiment 7	1	2(40%)	2(2.5)	2(68)	0.711
Experiment 8	1	3(50%)	3(3)	3(72)	0.734
Experiment 9	2(30%)	1	2	3	0.729
Experiment 10	2	2	3	1	0.710
Experiment 11	2	3	1	2	0.713
Experiment 12	3(40%)	1	3	2	0.723
Experiment 13	3	2	1	3	0.727
Experiment 14	3	3	2	1	0.697
Mean value 1	0.711	0.713	0.709	0.698	
Mean value 2	0.717	0.716	0.712	0.716	
Mean value 3	0.717	0.715	0.722	0.730	
Sample Range	0.006	0.003	0.013	0.032	

**Table 8:** The intuitive analysis table of stack consistency at 0.70 A cm^−2^.

Column	1	2	3	4	
Factor	RH_a_	RH_c_	Stoich. (c)	T (Inlet Water) (°C)	Standard Deviation (SD)
Experiment 6	1(20%)	1(30%)	1(2.0)	1(65)	0.017
Experiment 7	1	2(40%)	2(2.5)	2(68)	0.009
Experiment 8	1	3(50%)	3(3)	3(72)	0.002
Experiment 9	2(30%)	1	2	3	0.004
Experiment 10	2	2	3	1	0.016
Experiment 11	2	3	1	2	0.007
Experiment 12	3(40%)	1	3	2	0.009
Experiment 13	3	2	1	3	0.001
Experiment 14	3	3	2	1	0.013
Mean value 1	0.009	0.010	0.008	0.015	
Mean value 2	0.009	0.009	0.009	0.008	
Mean value 3	0.008	0.007	0.009	0.002	
Sample Range	0.001	0.003	0.001	0.013	

**Table 9:** The intuitive analysis table of stack performance at 1.20 A cm^−2^.

Column	1	2	3	4	
Factor	RH_a_	RH_c_	Stoich. (c)	T (Inlet Water) (°C)	Stack Performance (V)
Experiment 16	1(20%)	1(30%)	1(2)	1(68)	0.647
Experiment 17	1	2(40%)	2(2.5)	2(70)	0.674
Experiment 18	1	3(50%)	3(3)	3(72)	0.678
Experiment 19	2(30%)	1	2	3	0.670
Experiment 20	2	2	3	1	0.671
Experiment 21	2	3	1	2	0.666
Experiment 22	3(40%)	1	3	2	0.674
Experiment 23	3	2	1	3	0.668
Experiment 24	3	3	2	1	0.667
Mean value 1	0.666	0.664	0.66	0.66	0.662
Mean value 2	0.669	0.671	0.67	0.67	0.671
Mean value 3	0.67	0.67	0.674	0.674	0.672
Sample Range	0.004	0.007	0.014	0.014	0.01

**Table 10:** The intuitive analysis table of stack consistency at 1.20 A cm^−2^.

Column	1	2	3	4	
Factor	RH_a_	RH_c_	Stoich. (c)	T (Inlet Water) (°C)	Standard Deviation (SD)
Experiment 16	1(20%)	1(30%)	1(2)	1(68)	0.013
Experiment 17	1	2(40%)	2(2.5)	2(70)	0.002
Experiment 18	1	3(50%)	3(3)	3(72)	0.002
Experiment 19	2(30%)	1	2	3	0.001
Experiment 20	2	2	3	1	0.005
Experiment 21	2	3	1	2	0.004
Experiment 22	3(40%)	1	3	2	0.001
Experiment 23	3	2	1	3	0.004
Experiment 24	3	3	2	1	0.004
Mean value 1	0.006	0.005	0.007	0.007	
Mean value 2	0.003	0.004	0.002	0.002	
Mean value 3	0.003	0.003	0.003	0.002	
Sample Range	0.003	0.002	0.005	0.005	

**Table 11:** The intuitive analysis table of stack performance at 1.60 A cm^−2^.

Column	1	2	3	4	
Factor	RH_a_	RH_c_	Stoich. (c)	T (Inlet Water) (°C)	Stack Performance (V)
Experiment 26	1(20%)	1(30%)	1(1.8)	1(68)	0.612
Experiment 27	1	2(40%)	2(2)	2(70)	0.626
Experiment 28	1	3(50%)	3(2.2)	3(72)	0.635
Experiment 29	2(30%)	1	2	3	0.631
Experiment 30	2	2	3	1	0.630
Experiment 31	2	3	1	2	0.618
Experiment 32	3(40%)	1	3	2	0.630
Experiment 33	3	2	1	3	0.625
Experiment 34	3	3	2	1	0.615
Mean value 1	0.624	0.624	0.618	0.619	
Mean value 2	0.626	0.627	0.624	0.625	
Mean value 3	0.623	0.623	0.632	0.630	
Sample Range	0.003	0.004	0.014	0.011	

**Table 12:** The intuitive analysis table of stack consistency at 1.60 A cm^−2^.

Column	1	2	3	4	
Factor	RH_a_	RH_c_	Stoich. (c)	T (Inlet Water) (°C)	Standard Deviation (SD)
Experiment 26	1(20%)	1(30%)	1(1.8)	1(68)	0.012
Experiment 27	1	2(40%)	2(2)	2(70)	0.010
Experiment 28	1	3(50%)	3(2.2)	3(72)	0.006
Experiment 29	2(30%)	1	2	3	0.004
Experiment 30	2	2	3	1	0.005
Experiment 31	2	3	1	2	0.009
Experiment 32	3(40%)	1	3	2	0.008
Experiment 33	3	2	1	3	0.008
Experiment 34	3	3	2	1	0.010
Mean value 1	0.009	0.008	0.010	0.009	
Mean value 2	0.006	0.008	0.008	0.009	
Mean value 3	0.009	0.008	0.006	0.006	
Sample Range	0.003	0.000	0.004	0.003	

**Table 13:** Optimal test parameters of the stack at different current densities.

Current Density (A cm^−2^)	Pressure (a/c, kPa)	RH_a_%	RH_c_%	Stoich(a)	Stoich(c)	T (Water Inlet) (°C)
0.30	60/50	20	40	1.8	4	65
0.70	85/70	40	40	1.5	3	72
1.20	100/80	40	40	1.5	3	72
1.60	120/100	30	40	1.5	2.2	72

## Data Availability

The original contributions presented in the study are included in the article, further inquiries can be directed to the corresponding author/s.
